# Bidirectional relationship of diabetic retinopathy with anxiety and depression: a meta-analysis

**DOI:** 10.3389/fendo.2026.1764745

**Published:** 2026-04-13

**Authors:** Seng Fan, Ziyue Gai, Hui Ma, Ziye Wen, Chunlei Ma, Dongying Fan, Songying Qian, Zengxin Li

**Affiliations:** 1 School of Nursing, Beihua University, Jilin, China; 2 Department of Nursing, Air Force Medical University, Xi’an, Shaanxi, China; 3 Nursing Department, Beihua University Affiliated Hospital, Jilin, China; 4 Endocrinology Department, Beihua University Affiliated Hospital, Jilin, China

**Keywords:** anxiety, depression, diabetes complications, diabetes mellitus, diabetic retinopathy, meta-analysis

## Abstract

**Introduction:**

Diabetic retinopathy (DR) stands as the most frequently observed microvascular complication caused by diabetes mellitus (DM). This study aimed to prove whether there was a bidirectional relationship of DR with anxiety and depression.

**Methods:**

This study included 34 studies in total. Two researchers independently screened and extracted the data relevant to this study. The Newcastle-Ottawa Scale and the tools of the Agency for Healthcare Research and Quality were employed for the evaluation of the included studies. Stata 15.1 was applied for computing the odds ratio (OR), hazard ratio (HR), and 95% confidence interval (CI), and the relationship of DR with anxiety and depression was analyzed. The publication bias was evaluated leveraging funnel plots and Egger’s test. The sensitivity analysis was conducted by sequentially removing each study.

**Results:**

This meta-analysis included 34 studies in total, involving 383,195 patients. A significantly positive correlation was observed between DR and depression (OR = 1.58; 95%CI:1.24-2.02; p<0.001). A significantly positive correlation was found between depression and DR (OR = 2.13; 95%CI:1.53-2.98; p<0.001). A significantly positive correlation existed between DR and anxiety (OR = 2.23; 95%CI:1.07-4.68; p=0.033).

**Conclusion:**

This study suggests a bidirectional relationship exists between DR and depression. Therefore, it is recommended to carry out personalized psychological care during the treatment of DR.

**Systematic Review Registration:**

https://www.crd.york.ac.uk/PROSPERO/view/CRD420251080025, identifier CRD420251080025.

## 1 Introduction

As one of the microvascular complications caused by diabetes mellitus (DM), diabetic retinopathy (DR) also stands as one of the most common factors causing blindness and visual impairment in adults of working age (1). Its pathological changes mainly include proliferation of capillary endothelial cells, thickening of basement membranes, and selective loss of pericytes. Those changes eventually trigger microaneurysms, increased microvascular permeability, disruption of the blood-retina barrier, capillary occlusion, and neovascularization (2–4). In clinical practices, DR is classified into non-proliferative DR and proliferative DR according to the severity of DR and the presence or absence of the neovascularization of abnormal retinas (5–7). Currently, many therapies can prevent the visual impairment caused by DR, including laser photocoagulation, intravitreal injection of anti-vascular endothelial growth factor (VEGF) drugs and corticosteroids, and vitrectomy (8). According to the latest research, the prevalence of DR among patients with DM is 22.27% (9). With the increasing global prevalence of DM, the prevalence of DR is also on the rise (10). In 2020, adult patients suffering from DR worldwide reached approximately 103 million (11), and it is estimated that by 2030, 191 million people around the globe will suffer from DR (12). Such a large patient population is at risk of vision loss or even blindness. Because long-term or even lifelong monitoring and treatment are required for DR, patients tend to suffer from negative psychological states, including anxiety and depression, during the long period of treatment. A study has revealed that the prevalence of depression and anxiety in patients suffering from DR is 34.3% and 41.1%, respectively (13). Anxiety and depression may lead to poorer control of blood glucose, a decrease in quality of life, and lower treatment compliance among patients with DR (14). Therefore, patients suffering from DR need particular attention.

Previous research (15) has demonstrated that there exists a bidirectional relationship of DM with depression. Nevertheless, the bidirectional relationship of DR with anxiety and depression remains unclear. Some studies suggested that DR could cause anxiety or depression, while others suggested that anxiety or depression could lead to DR. Therefore, the bidirectional relationship of DR with anxiety and depression is controversial.

Current meta-analyses (16, 17) demonstrate a positive correlation between depression and DR. Some studies (18–21) suggest a positive correlation between DR and depression. No correlation between DR and depression is found in the study by C. Chen et al. (22). Therefore, it is essential to conduct this study to comprehensively analyze the bidirectional relationship of DR with anxiety and depression to update and supplement existing research. This study may help to provide a scientific basis for revealing the vicious cycle mechanism of DR between anxiety and depression being mutually causal, and help to support clinical medical staff to formulate comprehensive prevention and treatment regimens, thereby enhancing the quality of life as well as the long-term prognosis of individuals with DR.

## 2 Methods

This study abided by the Preferred Reporting Items for Systematic reviews and Meta-Analyses (PRISMA) (23), with the study’s protocol registered on the Prospective Register of Systematic Reviews (PROSPERO) (CRD420251080025).

### 2.1 Search strategy for literature

Databases, comprising Embase, PubMed, Cochrane Library, and Web of Science, were searched for target literature since their establishment up to June 21, 2025. The language of the target literature was English. The search was conducted using combinations of subject terms with free terms. The medical subject terms used were ‘diabetic retinopathy’ and ‘depression or anxiety’. Additionally, manual searches were conducted of the references in other related literature and grey literature to find studies meeting the criteria. The detailed search strategy is shown in **Supplementary Table 1**.

### 2.2 Inclusion and exclusion criteria

The literature satisfying the criteria below was included in this study (1): Study subject: patients with DR and depression or anxiety caused by DM; (2) Exposure group: DR and depression or anxiety; Control group: non-DR, non-depression, or non-anxiety; (3) Study type: observational study; (4) Outcome indicators: the relationship between DR and anxiety or depression.

Literature with the following features was excluded: (1) Animal or cell experiment, case report, science experiment project, review, letter, editorial, and conference paper; (2) Literature with missing or critically erroneous data; (3) Duplicate literature; (4) Literature with no full texts; (5) Literature with duplicate participants.

### 2.3 Extraction of data

The searched literature was loaded into EndNote. Two researchers (Seng Fan and Ziyue Gai) screened the titles and abstracts of the literature independently on the basis of the inclusion and exclusion criteria, and then reviewed the full texts. Regarding the controversial literature, discussions or seeking the advice of a third researcher (Zengxin Li) were needed before re-evaluation. The data extraction was carried out independently by the 2 researchers, leveraging a pre-designed spreadsheet, comprising first author, publication year, study design, region, number of cases, sex (male/female), age, relationship direction, outcome indicator, and quality score.

### 2.4 Quality assessment

Two investigators (Seng Fan and Ziyue Gai) independently employed the Newcastle-Ottawa Scale (NOS) (24) to assess the quality of the included studies. It consists of 8 items, comprising 3 dimensions of selection, comparability, and exposure. Specifically, the quality score of NOS ranged from 0 to 9, and studies scoring 6 or above were rated as studies of high quality, while those scoring 5 or below were rated as studies of low quality.

For the quality assessment of cross-sectional studies, 11 items suggested by the Agency for Healthcare Research and Quality (AHRQ) were adopted. A score of 0 to 3 meant low quality, 4 to 7 meant medium quality, and 8 to 11 meant high quality.

### 2.5 Statistical analysis

The statistical analysis was conducted leveraging STATA 15.1. The bidirectional relationship between DR and depression was evaluated by calculating the odds ratio (OR), hazard ratio (HR), and 95% confidence interval (CI). Heterogeneity was evaluated using the Cochrane I^2^ statistics. A P-value < 0.10 and an I^2^ > 50% were considered to indicate statistical heterogeneity. If significant heterogeneity was observed, a random-effects model was adopted, and a fixed-effects model was chosen otherwise. Sensitivity analysis was employed to evaluate the robustness of the results. Subgroup analysis (confounding factors, study design, region) and meta-regression were employed to determine the sources of heterogeneity. For outcomes with more than 10 studies, the publication bias was assessed using funnel plots and was quantified leveraging Egger’s test. A P-value < 0.05 indicated significant bias.

## 3 Results

### 3.1 Process of literature search and screening

A total of 4,586 records in total were found, and 806 duplicate records were excluded. Following the initial review of the titles and abstracts, 3,676 records were removed. The full texts of the rest literature were reviewed, and finally, 34 studies in total were included on the basis of the strict inclusion and exclusion criteria. The specific screening process is illustrated in **Supplementary Figure 1**.

### 3.2 Characteristics and quality assessment of the included studies

The 34 included studies (25–58) covered 14 countries (America, Australia, China, Denmark, Germany, India, Iran, Japan, Saudi Arabia, Lebanon, Pakistan, Tanzania, Turkey, and the United Kingdom), involving a total of 383,195 patients. Among them, 218,477 were males, and 164,718 were females, aged from 47.2 to 73.4 years. The characteristics of the included studies are expressed in **Table 1**.

**Table 1:** The basic characteristics and quality assessment of the included studies.

First author	Publication year	Study design	Region	Number of cases	Sex (male/female)	Age	Relationship direction	Outcome indicator	Quality score
A. A. Al-Ghamdi	2004	Cross-sectional	Saudi Arabia	200	77/123	52 ± 13.6	B	OR	ARRQ=8
H.-J. Lee	2009	Cross-sectional	American	55	32/23	58.9 ± 9.99	B	OR	ARRQ=7
T. T. Nguyen	2010	Case-control	Australia	92	28/64	60.55 ± 10.68	B	OR	NOS=8
Z. A. Saglam	2010	Cohort	Turkey	394	160/234	51.43 ± 11.51	A	OR	NOS=8
N. Sieu, W. Katon	2011	Cohort	American	2355	1230/1125	63.9 ± 12.9	B	OR HR	NOS=8
F. E. Hirai	2012	Cross-sectional	American	484	235/249	49.1 ± 9.25	B	OR	ARRQ=9
N. Ali	2013	Cross-sectional	India	122	60/62	47.2 ± 9.4	B	OR	ARRQ=7
K. Ishizawa	2016	Cross-sectional	Japan	4283	2626/1657	73.4 ± 6.0	B	OR	ARRQ=10
R. Rajput	2016	Case-control	India	410	197/213	54.73 ± 9.9	A C	OR	NOS=7
G. Rees	2016	Cross-sectional	Australia	519	349/170	64.9 ± 11.6	A	OR	ARRQ=10
P. Bahety	2017	Case-control	India	100	48/52	56.09 ± 5.92	A	OR	NOS=6
K. Ismail	2017	Cohort	United Kingdom	1651	909/742	56.21 ± 11.06	B	OR	NOS=8
N. Prinz	2017	Cohort	Germany	17563	9748/7815	63.8 ± 11.27	B	OR	NOS=8
H. Ahmadieh	2018	Cross-sectional	Lebanon	436	156/280	64.08 ± 17.06	B	OR	ARRQ=9
S. Sharif	2019	Cross-sectional	Pakistan	100	45/55	58.3 ± 12.4	A	OR	ARRQ=8
J. M. Bloom	2020	Cross-sectional	American	74	31/43	65.34 ± 12.57	C	OR	ARRQ=6
N. Khodabandehloo	2020	Cross-sectional	Iran	100	40/60	NA	B	OR	ARRQ=8
W. Aljohani	2021	Cross-sectional	Saudi Arabia	267	172/95	57.88 ± 8.71	A	OR	ARRQ=8
S. Pal	2021	Cross-sectional	India	290	155/135	58.2 ± 11.08	B	OR	ARRQ=8
D. Simayi	2022	Cross-sectional	China	874	536/338	59.08 ± 12.27	B	OR	ARRQ=8
X. J. Sun	2022	Cross-sectional	American	935	519/416	61.0 ± 1.3	A	OR	ARRQ=10
J. Vidyulatha	2022	Cross-sectional	India	315	186/129	50 ± 12	A C	OR	ARRQ=9
Y. K. Bao	2023	Cross-sectional	American	355	190/165	56.66 ± 29.06	A	OR	ARRQ=9
Z. Lyu	2023	Cross-sectional	American	669	369/300	56.3 ± 0.38	A	OR	ARRQ=8
M. R. Mussa	2023	Cross-sectional	Tanzania	267	142/125	50 ± 14	A	OR	ARRQ=9
F. N. Pedersen	2023	Cohort	Denmark	240893	136998/103895	65.24 ± 12.44	A	OR HR	NOS=7
G. Valluru	2023	Cross-sectional	American	5495	2748/2747	NA	A	OR	ARRQ=9
T. A. Horsbol	2024	Cohort	Denmark	66123	43287/22836	NA	A C	HR	NOS=8
P. Kalva	2024	Cross-sectional	American	664	367/297	63.0 ± 12.4	A	OR	ARRQ=9
B. Li	2024	Cross-sectional	American	767	407/360	51.47 ± 9.43	A	OR	ARRQ=8
A. G. Hamedani	2025	Cohort	American	10089	4205/5884	NA	A C	OR	NOS=8
M. T. H. Ho	2025	Cohort	China	12206	3648/8558	61.8 ± 13.4	B	HR	NOS=4
G. Li	2025	Cohort	UK	13706	8365/5341	60.11 ± 7.44	B	HR	NOS=7
J. Li	2025	Cross-sectional	American	342	212/130	59.51 ± 0.94	A	OR	ARRQ=8

A, Prediction of depression by DR; B, Prediction of DR by depression; C, Prediction of anxiety by DR.

OR, Odds ratio; HR, Hazard ratio; NOS, the Newcastle-Ottawa Scale; AHRQ, the Agency for Healthcare Research and Quality.

Of the 34 studies included, 22 cross-sectional studies (25, 26, 30–32, 34, 38–49, 51, 53, 54, 58) had a quality score higher than 7, suggesting they were all medium to high-quality studies; 3 pathological report studies (27, 33, 35) demonstrated a quality score higher than 6, suggesting they were all high-quality studies; 9 cohort studies (28, 29, 36, 37, 50, 52, 55–57) reached a quality score higher than 7, suggesting they were all high-quality studies. However, the study by M. T. H. Ho (56) had a low-quality score and did not describe the determination of exposure, comparability, and methods of result measurement.

### 3.3 Results of the meta-analysis

#### 3.3.1 Prediction of depression by DR

##### 3.3.1.1 Odds ratio

**Figure 1:**
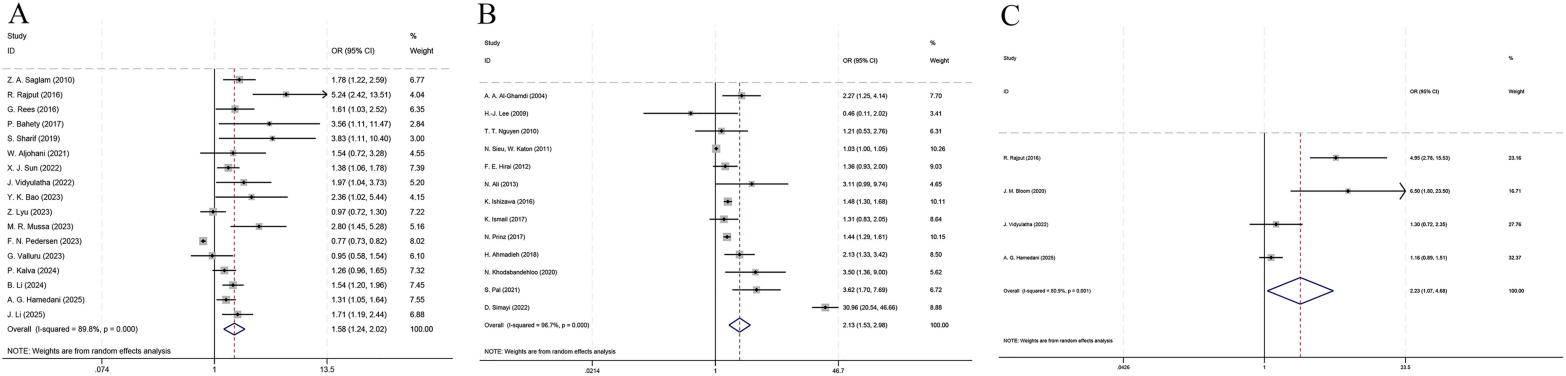
Forest plot of the odds ratio for diabetic retinopathy and depression or anxiety. **(A)** prediction of depression by diabetic retinopathy; **(B)** prediction of diabetic retinopathy by depression; **(C)** prediction of anxiety by diabetic retinopathy.

A total of 17 studies involving 262,581 patients were analyzed for this outcome (**Figure 1A**). The heterogeneity analysis showed an I^2^ = 89.8% and a P < 0.001, and thus the random-effects model was utilized.

The pooled results (**Figure 1A**) indicated that among patients with DM, the risk of depression among patients suffering from DR was significantly higher than that among patients without DR (OR = 1.58, 95% CI: 1.24 - 2.02, p < 0.001).

##### 3.3.1.2 Subgroup analysis

To determine the source of heterogeneity, subgroup analyses were conducted on the included population by confounding factors, study design, and region. The results are presented in **Table 2**. In the subgroup analysis by confounding factors, it was found that the results of both 2 groups were significant, suggesting that DR was a risk factor for depression among patients with DM. The subgroup analysis by study design suggested that the results of the cohort group were not significant, and the intra-group heterogeneity of the case-control group (P = 0.602, I2 = 0.0%) and the cross-sectional group (P = 0.03, I2 = 48.5%) significantly decreased, indicating that study design might be a source of heterogeneity. In the subgroup analysis by region, it was found that the results of the Asia group and the North America group were significant, while the results of the Oceania group were not significant. Only one study was in the Africa group, and its heterogeneity could not be determined. Therefore, the study design could not be determined as a source of heterogeneity.

**Table 2:** Subgroup analysis of diabetic retinopathy predicting incident depression.

Subgroup	Number of studies	I^2^ (%)	Heterogeneity p-value	OR	95%CI	Z	P
Confounding factors							
NO	8	69.2	0.002	1.79	1.32-2.44	3.71	<0.001
YES	9	90.0	<0.001	1.39	1.02-1.88	2.11	0.035
Study design							
Cohort	3	94.7	<0.001	1.19	0.72-1.96	0.66	0.507
Case-control	2	0	0.602	4.58	2.29-9.15	4.30	<0.001
Cross-sectional	12	48.5	0.030	1.47	1.24-1.75	4.45	<0.001
Region							
Asia	6	34.7	0.176	2.33	1.61-3.37	4.49	<0.001
Oceania	2	90.2	0.001	1.07	0.52-2.20	0.20	0.845
North America	8	39.4	0.116	1.32	1.15-1.52	3.85	<0.001
Africa	1	–	–	2.80	1.47-5.34	3.12	<0.001

##### 3.3.1.3 Hazard ratio

**Figure 2:**
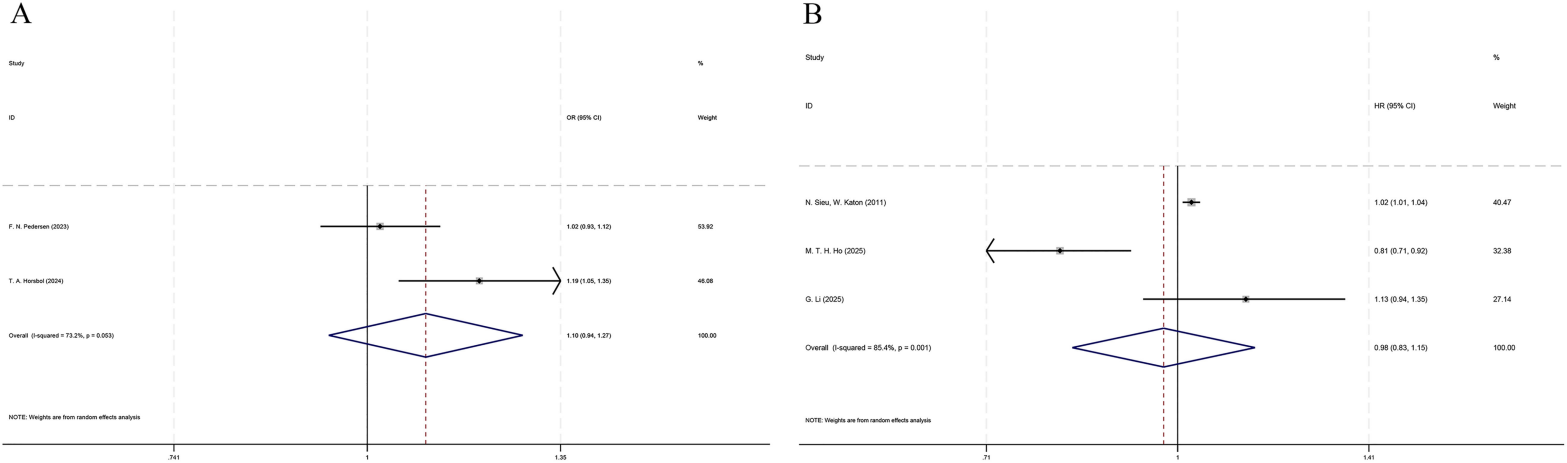
Forest plot of the hazard ratio for diabetic retinopathy and depression or anxiety. **(A)** prediction of depression by diabetic retinopathy; **(B)** prediction of diabetic retinopathy by depression.

A total of 2 studies with 307,016 patients involved were analyzed for this outcome (**Figure 2A**). The heterogeneity analysis showed an I^2^ = 73.2% and a P = 0.053. As a result, the random-effects model was selected.

Among patients with DM, no statistically significant difference was observed between patients with DR and those without DR in terms of the risk of depression (HR = 1.10, 95% CI: 0.94 - 1.27, p = 0.237).

#### 3.3.2 Prediction of DR by depression

##### 3.3.2.1 Odds ratio

A total of 13 studies covering 28,505 patients were analyzed for this outcome (**Figure 1B**). The heterogeneity analysis indicated an I^2^ = 96.7% and a P < 0.01; hence, the random-effects model was employed.

The pooled results (**Figure 1B**) suggested that among patients with DM, the risk of DR in the depression group was significantly higher than that in the non-depression group (OR = 2.13, 95% CI: 1.53 - 2.98, p < 0.01).

##### 3.3.2.2 Subgroup analysis

To determine the source of heterogeneity, subgroup analyses were conducted on the included population by confounding factors, study design, and region. **Table 3** expresses the results. In the subgroup analysis by confounding factors, the results of the NO group were significant, while those of the YES group were not significant. After the control for confounding factors, depression might not be a risk factor for DR. However, only two studies were in the YES group. As a result, cautious interpretation of the results was essential. In the subgroup analysis by study design, the results of the cross-sectional group were significant, while those of the cohort group were not significant. Only one study was in the case-control group, and its heterogeneity could not be determined. Therefore, the study design could not be considered as the source of heterogeneity. In the subgroup analysis by region, the results of the Asia group and the Europe group were significant, while those of the North America group were not significant. Only one study was in the Oceania group, and its heterogeneity could not be determined. As a result, the study design could not be thought of as a source of heterogeneity.

**Table 3:** Subgroup analysis of depression predicting incident diabetic retinopathy.

Subgroup	Number of studies	I^2^ (%)	Heterogeneity p-value	OR	95%CI	Z	P
Confounding factors							
NO	11	95.2	<0.001	2.37	1.24-4.53	2.62	0.009
YES	2	97.1	<0.001	1.21	0.87-1.69	1.13	0.260
Study design							
Cohort	3	94.3	<0.001	1.23	0.92-1.64	1.41	0.160
Case-control	1	–	–	1.21	0.53-2.76	0.46	0.646
Cross-sectional	9	96.1	<0.001	2.73	1.25-5.96	2.51	0.012
Region							
Asia	7	96.9	<0.001	3.67	1.35-10.00	2.54	0.011
Oceania	1	–	–	1.21	0.53-2.76	0.46	0.646
North America	3	37.8	0.200	1.08	0.85-1.38	0.63	0.532
Europe	2	0	0.685	1.43	1.29-1.59	6.54	<0.001

##### 3.3.2.3 Hazard ratio

A total of 3 studies covering 28,267 patients were analyzed for this outcome (**Figure 2B**). The heterogeneity analysis showed an I^2^ = 85.4% and P = 0.001; therefore, the random-effects model was chosen.

Among patients with DM, no statistically significant difference was noticed between the depression group and the non-depression group in terms of the risk of DR (HR = 0.98, 95% CI: 0.83 - 1.15, p = 0.764).

#### 3.3.3 Prediction of anxiety by DR

A total of 4 studies with 10,890 patients involved were analyzed for this outcome (**Figure 1C**). The heterogeneity analysis showed an I^2^ = 80.9% and a P = 0.001. Hence, the random-effects model was employed.

The pooled results (**Figure 1C**) demonstrated that among patients with DM, the risk of anxiety in the DR group was significantly higher than that in the non-DR group (OR = 2.23, 95% CI: 1.07 - 4.68, p = 0.033).

##### 3.3.3.1 Subgroup analysis

Subgroup analyses were performed on the included population by confounding factors, study design, and region. The results are presented in **Table 4**. In the subgroup analysis by region, it was found that the results of both 2 groups were not significant, suggesting that region was not the source of heterogeneity. In the subgroup analysis by confounding factors, the results of the NO group were significant, while those of the YES group were not significant. After the control for confounding factors, DR might not be a risk factor for anxiety. In the subgroup analysis by study design, the results of the cross-sectional group were not significant, and there was only one study in the cohort and case-control group, preventing the assessment of heterogeneity. Therefore, the study design could not be a source of heterogeneity.

**Table 4:** Subgroup analysis of diabetic retinopathy predicting incident anxiety.

Subgroup	Number of studies	I^2^ (%)	Heterogeneity p-value	OR	95%CI	Z	P
Confounding factors							
NO	2	0	0.730	5.39	2.63-11.02	4.61	<0.001
YES	2	0	0.730	1.18	0.93-1.50	1.36	0.174
Study design							
Cohort	1	–	–	1.16	0.89-1.51	1.10	0.271
Case-control	1	–	–	4.95	2.09-11.71	3.64	<0.001
Cross-sectional	2	79.9	0.026	2.62	0.55-12.50	1.21	0.228
Region							
Asia	2	84.1	0.012	2.44	0.66-9.04	1.34	0.181
North America	2	84.9	0.010	2.44	0.46-12.98	1.04	0.297

### 3.4 Results of the sensitivity analysis

For the evaluation of the robustness of the meta-analysis results, a sensitivity analysis was conducted. Each study was sequentially excluded, and the changes in the effect values were evaluated after the exclusion of certain studies. No significant changes were found, and the results were stable (**Supplementary Figure 2A**). In the sensitivity analysis of depression predicting DR, the study by D. Simayi et al. (44) was excluded, and then, a significant change in the effect value was observed. Therefore, their study might be one of the sources of heterogeneity (**Supplementary Figure 2B**). Due to the influence of this study, the sensitivity analysis results for depression predicting DR might be less stable.

### 3.5 Publication bias

To assess the publication bias in the meta-analysis, the funnel plot and the Egger’s test were employed for the outcomes with more than 10 studies.

The results (**Supplementary Figures 3A, B**) demonstrated that the funnel plots were asymmetrical for both the prediction of depression by DR and the prediction of DR by depression. Furthermore, based on Egger’s test results (p < 0.05), it was found that there might be publication bias. The trim-and-fill method was employed to correct for publication bias. Nevertheless, it was found that this method failed to correct the bias. Therefore, publication bias might impact the conclusions of this study, and more evidence is warranted to support the findings (**Supplementary Figures 4A, B**).

### 3.6 Meta-regression

Meta-regression was conducted on the outcomes (prediction of depression by DR and prediction of DR by depression) with more than 10 studies and significant heterogeneity to determine the potential sources of heterogeneity. It was found that in the outcome indicators of prediction of depression by DR, the regression results of case number (p = 0.021) and age (p = 0.013) were statistically significant, suggesting that case number and age were possible sources of heterogeneity in the results of prediction of depression by DR. In the outcome indicators of prediction of DR by depression, the regression result of publication year (p = 0.047) was statistically significant, suggesting that publication year was a possible source of heterogeneity in the results of prediction of DR by depression.

## 4 Discussion

This meta-analysis is the first to reveal a bidirectional relationship between DR and depression, while only a positive correlation is noted between DR and anxiety.

Current research has shown that anxiety and depression are associated with complications of DM (59, 60); however, most of them typically analyze complications of DM as a composite endpoint and pay less attention to DR (16). Therefore, this study first explored the bidirectional relationship of DR with anxiety and depression. The meta-analysis results revealed that DR possibly accelerated the progression of depression, which aligns with the conclusion of the cohort study by A. G. Hamedani et al. (55). Recent studies have revealed that a positive correlation is observed between DM duration and depression, indicating that individuals suffering from DR with a longer DM duration face a higher risk of developing depression (61). Furthermore, visual impairment and blindness caused by DR not only affect the lifestyle and work ability of a patient but also easily lead to psychological stress responses, which further trigger depressive symptoms (1, 21). It is worth noting that the literature included in this study primarily focused on middle-aged and older adults. Due to factors such as a decline in physical function, changes in hormone levels, and a shrinking of social networks, this population was significantly associated with a risk of depression (21). Conversely, this study also found that depression was a risk factor promoting the onset and progression of DR, which is consistent with the findings of the cohort study by G. Li et al. (57). Depression, through activating the hypothalamic-pituitary-adrenal axis and the sympathetic nervous system, causes elevated peripheral glucocorticoid and catecholamine levels, decreased insulin sensitivity, increased inflammation, and platelet aggregation, thereby leading to poor control of blood glucose and increasing the risk of DR (62, 63). Studies have shown that there is a correlation between healthy behaviors and depression control (64). Nevertheless, patients with DM accompanied by depression usually have unhealthy behaviors, such as smoking, excessive drinking, and an unhealthy diet, which are all risk factors for exacerbating the progression of DR (65–67). Additionally, this study revealed that DR might accelerate the progression of anxiety, which aligns with the conclusion of the cohort study by A. G. Hamedani et al. (55). During intravitreal anti-VEGF injections, patients with DR may experience a certain degree of pain. Existing studies have shown a positive correlation between pain level and anxiety among patients undergoing intravitreal anti-VEGF injections, meaning that patients with higher pain levels also exhibit higher anxiety levels. Furthermore, as the disease burden worsens in patients with advanced DR, their psychological stress increases accordingly, leading to a higher risk of anxiety (7, 8, 68, 69).

This study found that the OR for both DR predicting depression and depression forecasting DR was greater than the HR. The reason for this could be that the HR took into account the time to event occurrence, whereas the OR did not incorporate time-related factors. Therefore, as events accumulated over time, the OR was typically larger than the HR.

Studying the bidirectional relationship of DR with anxiety and depression shows many clinical implications in terms of enhancing the quality of life of patients, enhancing patient treatment compliance, and promoting the development of integrated care models. During clinical work, medical staff are supposed to pay more attention to the emotional changes of patients with DR and adopt personalized treatment regimens to prevent negative emotions such as anxiety or depression. Additionally, for patients with DM accompanied by depression, more comprehensive screening and early identification should be implemented to prevent DR and other complications of DM.

The strength of this study lies in the extensive literature search. It integrated the most comprehensive observational studies on DR and anxiety as well as depression up to now, filling the gap in the meta-analysis of the bidirectional relationship between DR and depression.

Nonetheless, certain limitations are also observed in this study. The majority of the studies included in this study consisted of cross-sectional studies, and this type of study design has inherent limitations in terms of causal inference. Therefore, it might, to a certain degree, impact the causal inference results of this study. Although confounders were adjusted for in this study, residual confounding may explain a substantial proportion of observed associations. The heterogeneity in the results of this study was relatively large. Although some sources of heterogeneity were determined through the subgroup analyses, sensitivity analyses, and meta-regression, these might not be able to explain all the heterogeneity. Furthermore, due to the small number of included studies in each subgroup analysis, the statistical power of the subgroup analysis results might have been affected, which, to some extent, further limited the reliability of the findings. Publication bias was observed in this meta-analysis, and its impact was not eliminated after correction using the trim-and-fill method. Accordingly, the credibility of the findings may remain compromised. Future research is supposed to include a larger number of high-quality cohort studies based on multi-center data and with a large sample size to more clearly explore the causal relationship between DR and depression.

## 5 Conclusion

In conclusion, this study indicates that a bidirectional relationship exists between DR and depression. In the future, more prospective studies are needed to further explore whether there exists a bidirectional relationship between DR and anxiety and to further investigate the causal relationship between DR and depression. This study may provide important support for the prevention and treatment of patients with DR and patients with DM accompanied by depression.

## Data Availability

The original contributions presented in the study are included in the article/**Supplementary Material**. Further inquiries can be directed to the corresponding author.
