# Supplementary material for: Bidirectional relationship of diabetic retinopathy with anxiety and depression: a meta-analysis
Source: Front Endocrinol (Lausanne). 2026 Apr 13;17:1764745. doi: 10.3389/fendo.2026.1764745 (PMC13111166; doi:10.3389/fendo.2026.1764745)
Supplement: Supplementary file 1 [file DataSheet1.docx]

**Table S1.** Search strategy

**The search strategy (Pubmed)**

| Search number | Query | Results |
| --- | --- | --- |
| 1 | diabetic retinopathy[MeSH Terms] | 32,234 |
| 2 | "diabetes mellitus retinopathy"[Title/Abstract] OR "diabetes retinopathy"[Title/Abstract] OR "diabetic retinitis"[Title/Abstract] OR "diabetic retinopathies"[Title/Abstract] OR "diabetic retinopathy"[Title/Abstract] OR "retinopathia diabetica"[Title/Abstract] OR "retinopathy in diabetes"[Title/Abstract] | 34,438 |
| 3 | (anxiety[MeSH Terms]) OR (depression[MeSH Terms]) | 361,595 |
| 4 | "Angst"[Title/Abstract] OR "anxiety"[Title/Abstract] OR "Anxiousness"[Title/Abstract] OR "central depression"[Title/Abstract] OR "clinical depression"[Title/Abstract] OR "depression"[Title/Abstract] OR "depressive disease"[Title/Abstract] OR "depressive disorder"[Title/Abstract] OR "depressive episode"[Title/Abstract] OR "depressive illness"[Title/Abstract] OR "depressive personality disorder"[Title/Abstract] OR "depressive state"[Title/Abstract] OR "Depressive Symptom"[Title/Abstract] OR "Depressive Symptoms"[Title/Abstract] OR "depressive syndrome"[Title/Abstract] OR "depressivity"[Title/Abstract] OR "Emotional Depression"[Title/Abstract] OR "Hypervigilance"[Title/Abstract] OR "mental depression"[Title/Abstract] OR "Nervousness"[Title/Abstract] OR "parental depression"[Title/Abstract] OR "Social Anxieties"[Title/Abstract] OR "Social Anxiety"[Title/Abstract] | 685,289 |
| 5 | (#1 OR #2) AND (#3 OR #4) | 314 |

**The search strategy (embase)**

| Search number | Query | Results |
| --- | --- | --- |
| 1 | 'diabetic retinopathy'/exp | 65142 |
| 2 | 'diabetes mellitus retinopathy':ab,ti,kw OR 'diabetes retinopathy':ab,ti,kw OR 'diabetic retinitis':ab,ti,kw OR 'diabetic retinopathies':ab,ti,kw OR 'diabetic retinopathy':ab,ti,kw OR 'retinopathia diabetica':ab,ti,kw OR 'retinopathy in diabetes':ab,ti,kw | 50586 |
| 3 | 'anxiety'/exp OR 'depression'/exp | 982923 |
| 4 | 'angst':ab,ti,kw OR 'anxiety':ab,ti,kw OR 'anxiousness':ab,ti,kw OR 'central depression':ab,ti,kw OR 'clinical depression':ab,ti,kw OR 'depression':ab,ti,kw OR 'depressive disease':ab,ti,kw OR 'depressive disorder':ab,ti,kw OR 'depressive episode':ab,ti,kw OR 'depressive illness':ab,ti,kw OR 'depressive personality disorder':ab,ti,kw OR 'depressive state':ab,ti,kw OR 'depressive symptom':ab,ti,kw OR 'depressive symptoms':ab,ti,kw OR 'depressive syndrome':ab,ti,kw OR 'depressivity':ab,ti,kw OR 'emotional depression':ab,ti,kw OR 'hypervigilance':ab,ti,kw OR 'mental depression':ab,ti,kw OR 'nervousness':ab,ti,kw OR 'parental depression':ab,ti,kw OR 'social anxieties':ab,ti,kw OR 'social anxiety':ab,ti,kw | 973272 |
| 5 | #1 OR #2 | 73537 |
| 6 | #3 OR #4 | 1283214 |
| 7 | #5 AND #6 | 1192 |

**The search strategy (Cochrane Library)**

| Search number | Query | Results |
| --- | --- | --- |
| 1 | MeSH descriptor: [Diabetic Retinopathy] explode all trees | 1993 |
| 2 | (‘diabetes mellitus retinopathy’ OR ‘diabetes retinopathy’ OR ‘diabetic retinitis’ OR ‘diabetic retinopathies’ OR ‘diabetic retinopathy’ OR ‘retinopathia diabetica’ OR ‘retinopathy in diabetes’):ab,ti,kw | 5526 |
| 3 | MeSH descriptor: [Anxiety] explode all trees | 13365 |
| 4 | MeSH descriptor: [Depression] explode all trees | 19217 |
| 5 | (‘Angst’ OR ‘anxiety’ OR ‘Anxiousness’ OR ‘central depression’ OR ‘clinical depression’ OR ‘depression’ OR ‘depressive disease’ OR ‘depressive disorder’ OR ‘depressive episode’ OR ‘depressive illness’ OR ‘depressive personality disorder’ OR ‘depressive state’ OR ‘Depressive Symptom’ OR ‘Depressive Symptoms’ OR ‘depressive syndrome’ OR ‘depressivity’ OR ‘Emotional Depression’ OR ‘Hypervigilance’ OR ‘mental depression’ OR ‘Nervousness’ OR ‘parental depression’ OR ‘Social Anxieties’ OR ‘Social Anxiety’):ab,ti,kw | 158843 |
| 6 | (#1 OR #2) AND (#3 OR #4 OR #5) | 113 |

**The search strategy (Web of science)**

| Search number | Query | Results |
| --- | --- | --- |
| 1 | TS=((diabetes mellitus retinopathy) OR (diabetes retinopathy) OR (diabetic retinitis) OR (diabetic retinopathies) OR (diabetic retinopathy) OR (retinopathia diabetica) OR (retinopathy in diabetes)) and Preprint Citation Index (Exclude – Database) | 116632 |
| 2 | TS=((Angst) OR (anxiety) OR (Anxiousness) OR (central depression) OR (clinical depression) OR (depression) OR (depressive disease) OR (depressive disorder) OR (depressive episode) OR (depressive illness) OR (depressive personality disorder) OR (depressive state) OR (Depressive Symptom) OR (Depressive Symptoms) OR (depressive syndrome) OR (depressivity) OR (Emotional Depression) OR (Hypervigilance) OR (mental depression) OR (Nervousness) OR (parental depression) OR (Social Anxieties) OR (Social Anxiety)) and Preprint Citation Index (Exclude – Database) | 1805687 |
| 3 | #1 AND #2 and Preprint Citation Index (Exclude – Database) | 2967 |

**
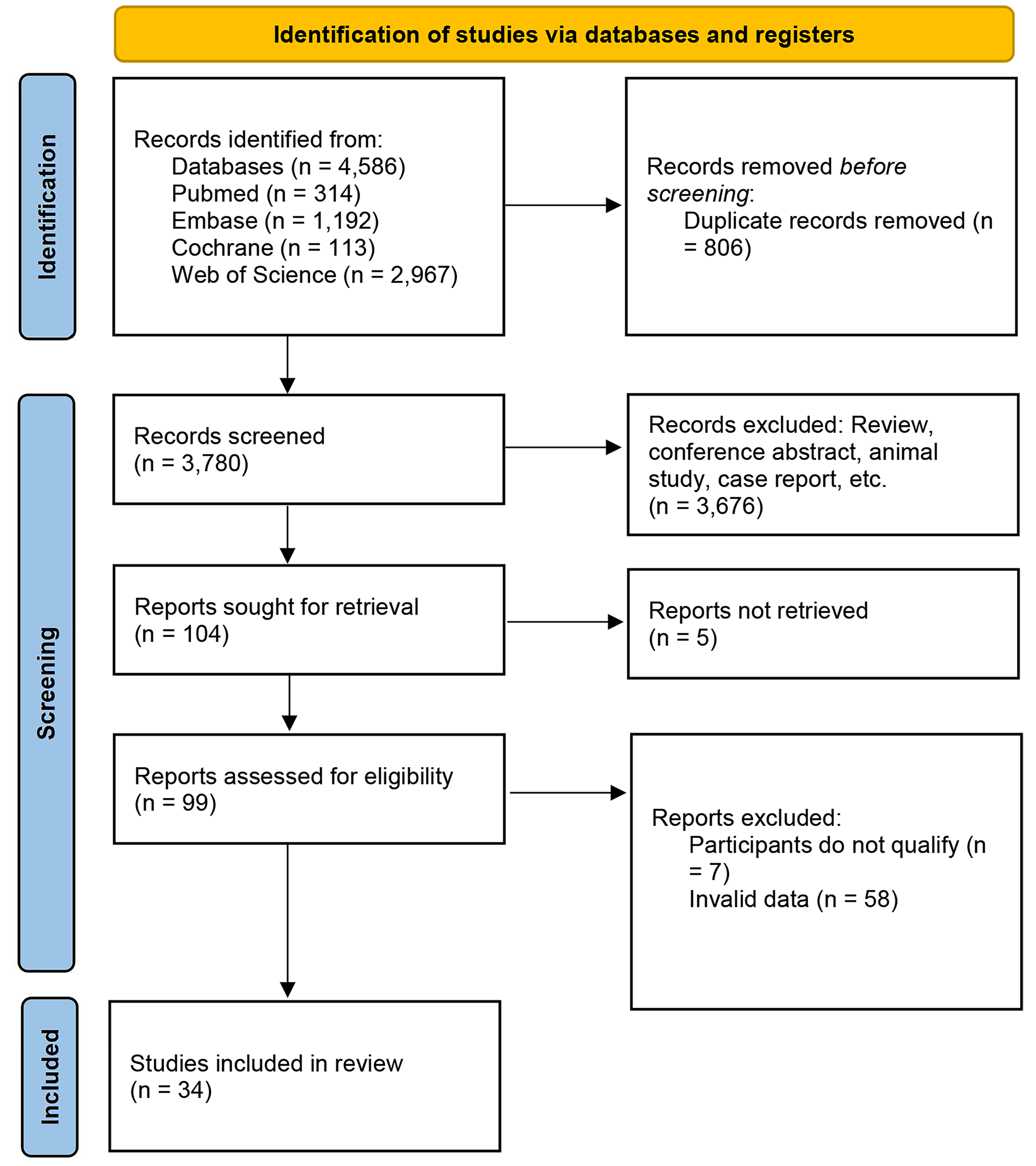
**

**Figure S1** Literature screening flow chart.


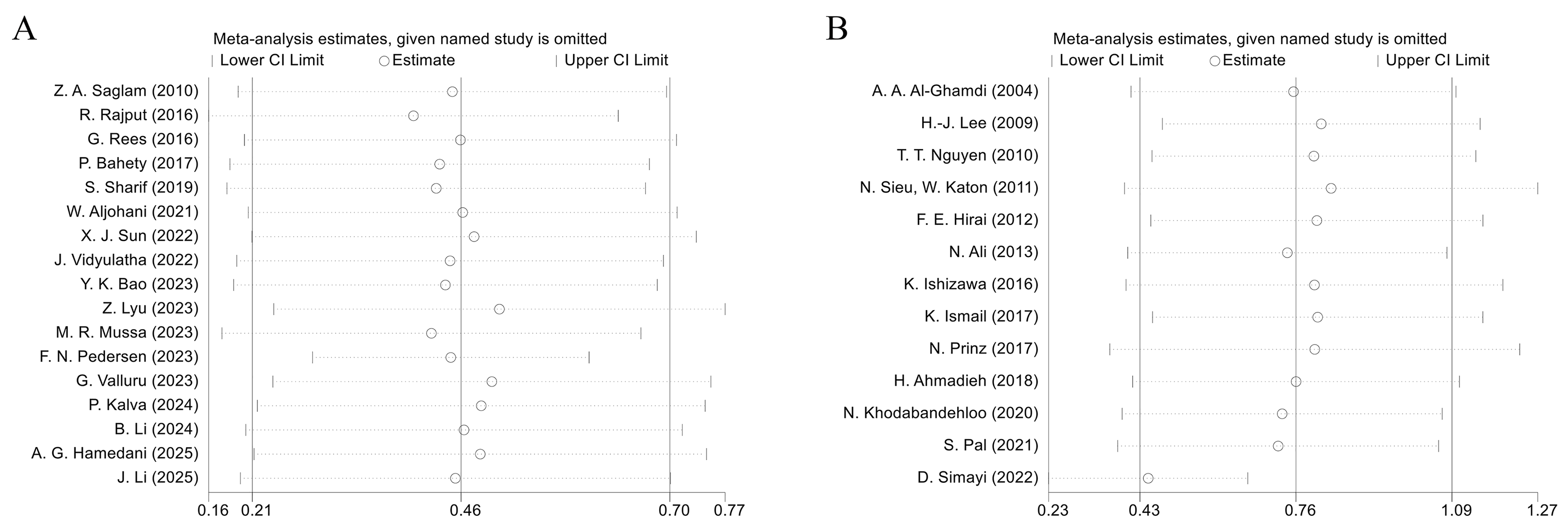


**Figure S2** Sensitivity analysis.

A: diabetic retinopathy predicting incident depression. B: depression predicting incident diabetic retinopathy.


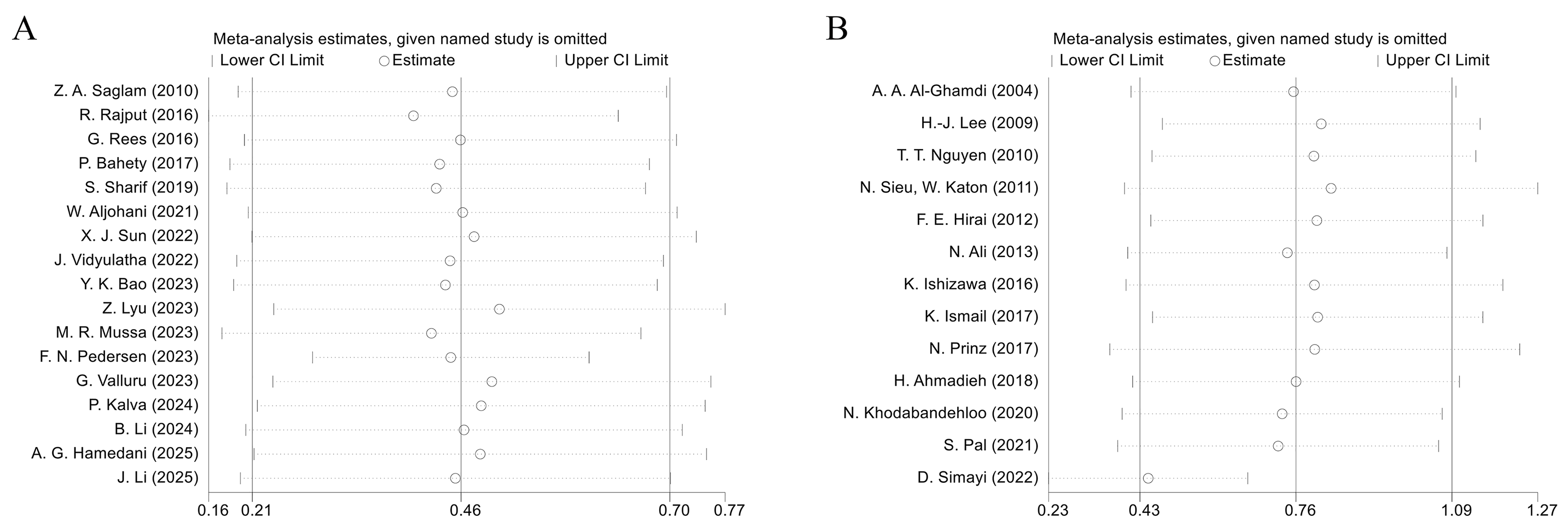


**Figures S3** Funnel plot.

A: diabetic retinopathy predicting incident depression. B: depression predicting incident diabetic retinopathy.


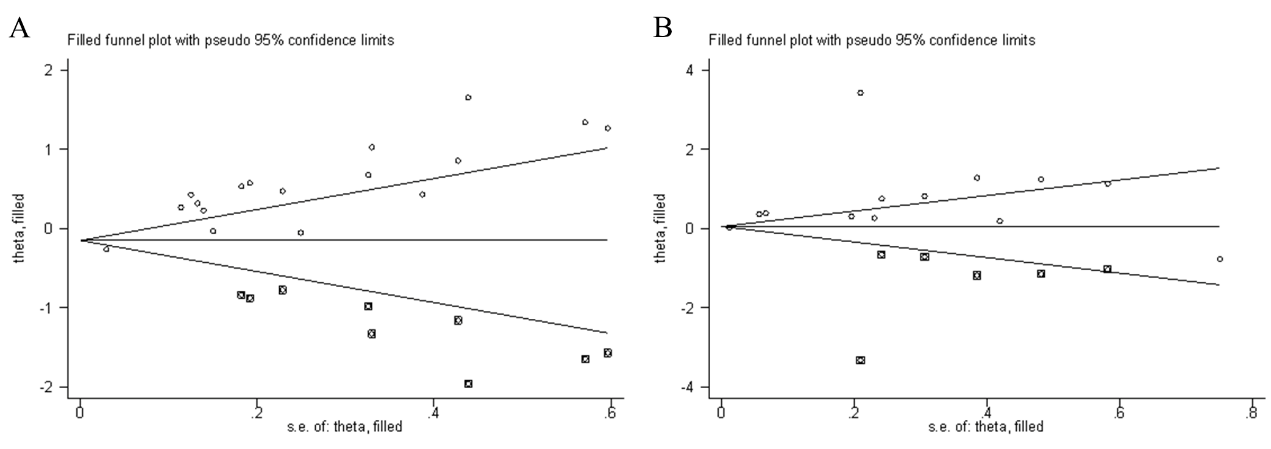


**Figure S4** Publication bias plot.

A: diabetic retinopathy predicting incident depression. B: depression predicting incident diabetic retinopathy.
